# Metabolic Reprogramming of Gastric Cancer Revealed by a Liquid Chromatography–Mass Spectrometry-Based Metabolomics Study

**DOI:** 10.3390/metabo15040222

**Published:** 2025-03-25

**Authors:** Lina Zhou, Benzhe Su, Zexing Shan, Zhenbo Gao, Xingyu Guo, Weiwei Wang, Xiaolin Wang, Wenli Sun, Shuai Yuan, Shulan Sun, Jianjun Zhang, Guowang Xu, Xiaohui Lin

**Affiliations:** 1 School of Computer Science and Technology, Dalian University of Technology, Dalian 116024, China; zhouln@dlut.edu.cn (L.Z.); benzhe.su.123@foxmail.com (B.S.); gaozb@mail.dlut.edu.cn (Z.G.); www123@mail.dlut.edu.cn (W.W.); 201987453@mail.dlut.edu.cn (W.S.); 2 CAS Key Laboratory of Separation Science for Analytical Chemistry, Dalian Institute of Chemical Physics, Chinese Academy of Sciences, Dalian 116023, China; guoxingyu@dicp.ac.cn (X.G.); wangxiaolin@dicp.ac.cn (X.W.); xugw@dicp.ac.cn (G.X.); 3 Instrumental Analysis Center, Dalian University of Technology, Dalian 116024, China; 4 Department of Gastric Surgery, Cancer Hospital of Dalian University of Technology (Liaoning Cancer Hospital & Institute), Shenyang 110042, China; shanzexing@cancerhosp-ln-cmu.com; 5 Central Laboratory, Cancer Hospital of Dalian University of Technology (Liaoning Cancer Hospital & Institute), Shenyang110042, China; yuanshuai@cancerhosp-ln-cmu.com (S.Y.); sunshulan@dlut.edu.cn (S.S.); 6 Liaoning Province Key Laboratory of Metabolomics, Dalian 116023, China

**Keywords:** gastric cancer, fatty acid β-oxidation, metabolic reprogramming, metabolomics

## Abstract

Background/Objectives: Gastric cancer (GC) is a prevalent malignant tumor worldwide, with its pathological mechanisms largely unknown. Understanding the metabolic reprogramming associated with GC is crucial for the prevention and treatment of this disease. This study aims to identify significant alterations in metabolites and pathways related to the development of GC. Methods: A liquid chromatography–mass spectrometry-based non-targeted metabolomics data acquisition was performed on paired tissues from 80 GC patients. Differences in metabolic profiles between tumor and adjacent normal tissues were first investigated through univariate and multivariate statistical analyses. Additionally, differential correlation network analysis and a newly proposed network analysis method (NAM) were employed to explore significant metabolite pathways and subnetworks related to tumorigenesis and various TNM stages of GC. Results: Over half of the annotated metabolites exhibited significant alterations. Phosphatidylcholine (PC)_30_0 and fatty acid C20_3 demonstrated strong diagnostic performance for GC, with AUCs of 0.911 and 0.934 in the discovery and validation sets, respectively. Differential correlation network analysis revealed significant fatty acid-related metabolic reprogramming in GC with elevated levels of medium-chain acylcarnitines and increased activity of medium-chain acyl-CoA dehydrogenase, firstly observed in clinical GC tissues. Of note, using NAM, two correlation subnetworks were identified as having significant alterations across different TNM stages, centered with choline and carnitine C4_0-OH, respectively. Conclusions: The identified significant alterations in fatty acid metabolism and TNM-related metabolic subnetworks in GC tissues will facilitate future investigations into the metabolic reprogramming associated with gastric cancer.

## 1. Introduction

Gastric cancer (GC) is a prominent malignant tumor worldwide, particularly prevalent in Asian countries [1], such as China [2]. *Helicobacter pylori* infection has been identified as a major risk factor for the development of GC [3,4]. Other contributing factors include smoking, gender, and dietary habits characterized by high salt and smoked foods [5]. Despite these associations, the precise pathological mechanisms underlying GC remain not well understood. After surgical resection, GC patients have high recurrence rates and poor overall survival [6]. The 5-year survival rate for early-stage GC, which is confined to the mucosa or submucosa, is higher than 90% after surgical treatment [7], while the 5-year survival rate for the advanced stage is only less than 10%. Many patients are diagnosed at an advanced stage, underscoring the urgent need for reliable biomarkers that enable earlier detection [8,9]. Thus, elucidating the biological events involving the occurrence and development of GC is crucial for enhancing prevention and treatment strategies.

Recent studies have deepened our understanding of the interplay between metabolic reprogramming and cancer [10,11,12,13,14]. Metabolomics provides the chance for the global analysis of metabolites. It provides critical insights into cancer states that may not be discernible through traditional methods [15]. However, metabolomic investigations of the GC tissue microenvironment remain limited [16,17]. Common metabolomics platforms include gas chromatography–mass spectrometry, liquid chromatography–mass spectrometry (LC-MS), capillary electrophoresis–mass spectrometry, and nuclear magnetic resonance spectroscopy. Each platform provides complementary metabolite information. Tissue, blood and urine are the most common biospecimen types. Relative to blood and urine, tissue metabolism can reflect the microenvironment more directly [18].

Dai et al. reported comparative analyses of the gastric microbiome and metabolome, which were conducted on 37 GC tumor tissues alongside matched non-tumor tissues, utilizing 16S rRNA gene sequencing and LC-MS metabolomics. These analyses identified a combinatorial biomarker consisting of 1-methylnicotinamide and N-acetyl-D-glucosamine-6-phosphate, capable of distinguishing GC tumors from non-tumor tissues. Furthermore, alterations in the metabolome of GC tumors may be partially attributable to the activities of *Helicobacter*, *Lactobacillus*, and other microbial species [19]. Kaji et al. explored the metabolic differences between paired cancerous and adjacent non-cancerous tissues from 140 GC patients via capillary electrophoresis-mass spectrometry, revealing that β-alanine serves as both a significant predictor of peritoneal recurrence and a prognostic marker [20]. Zhang et al. performed 1H NMR-based metabolic profiling, and the metabolic profiles of tissue samples from 40 lymph node metastasis (LNM)-positive GC patients, 40 LNM-negative GC patients, and 40 normal controls were compared. Leucine, isoleucine, valine, glutathione and betaine were found as potential diagnostic and prognostic factors for GC patients having LNM or not [21]. Several other studies have also explored metabolic alterations in GC tissues from clinical patients [22,23,24,25,26,27].

Despite the reported differential metabolites, there is a notable lack of consistency across existing metabolomic studies on GC. Variations in analytical platforms, experimental designs, and limited sample sizes likely contribute to these discrepancies [28]. Moreover, LC-MS-based metabolomic analyses of GC tissues are scarce, with small sample sizes prevalent in the literature [29]. Notably, few studies have systematically investigated the relationships between differential metabolites and clinical indices.

In this study, we employed LC-MS-based metabolomics to analyze tumor tissues and matched adjacent normal tissues from 80 patients with GC. Our objective was to characterize the metabolic reprogramming occurring within the tumor microenvironment. By integrating this metabolic data with a comprehensive collection of clinical information, the associations between differential metabolites and the clinical indices were further explored. Ultimately, this study seeks to discover key metabolic reprogramming of GC and elucidate the molecular mechanism underlying GC development.

## 2. Materials and Methods

### 2.1. Chemicals and Reagents

HPLC grade methanol and acetonitrile were obtained from Merck (Merck, Darmstadt, Germany). Internal standards were purchased from Sigma-Aldrich (St. Louis, MO, USA) and Cambridge Isotope Laboratories (Andover, MA, USA). Ultrapure water was purified with a Milli-Q system (Millipore, Billerica, MA, USA). Formic acid and ammonium bicarbonate of the HPLC grade were obtained from J&K Scientific Ltd. (Beijing, China).

### 2.2. Tissue Sample Collection and Preparation

Eighty GC patients were recruited for untargeted metabolomic analysis at the Liaoning Cancer Hospital and Institute. General anesthesia was provided before the operation. No enrolled patients underwent radiotherapy/chemotherapy before the operation. Paired cancer tissue and non-tumor tissue were collected from patients. The fresh tissue samples were rinsed with sterile water, frozen in liquid nitrogen, and transferred to −80 °C refrigerator for storage [19]. Non-tumor tissue samples were defined as gastric mucosal tissue > 5 cm away from the matched tumor tissue.

Being put on ice and approximately 10 mg of tissues were cut and transferred to 2 mL eppendorf tubes. Then, 600 μL of 80% methanol in water [30] containing internal standards was added to the tubes and homogenized using an MM400 mixer mill (Retsch, Haan, Germany) at 28 Hz, twice for 30 s. The internal standards at the final concentrations were as follows: 0.1 μg/mL carnitine C2:0-d3, 0.1 μg/mL carnitine C8:0-d3, 0.1 μg/mL carnitine C16:0-d3, 1 μg/mL LPC 19:0, 2 μg/mL fatty acid (FA), 16:0-d3 and 2 μg/mL FA 18:0-d3, 1 μg/mL chenodeoxycholic acid-d4, 1 μg/mL cholic acid-d4, 2 μg/mL tryptophan-d5, 2 μg/mL phenylalanine-d5, 0.1 μg/mL sphingomyelin 12:0, and 0.4 μg/mL choline-d4. After the centrifugation of 20,000× *g* for 10 min at 4 °C, a 400 μL aliquot of the supernatant was drawn and lyophilized. The dried samples were stored at −80 °C before the analysis. After being resuspended in 80 μL of 25% acetonitrile in water, 5 µL of the supernatant was injected for the LC-MS analysis.

### 2.3. LC-MS Analysis

For non-targeted LC-MS metabolomics analysis, a Shimadzu LC 40D X3 coupled to the SCIEX ZenoTOF 7600 (AB SCIEX, Framingham, MA, USA) system was used. For electrospray ionization positive (ESI+) and negative (ESI−) ionization modes, a 50 mm × 2.1 mm, 1.7 μm Waters BEH C8 (Waters, Milford, MA, USA) column, and a 50 mm × 2.1 mm, 1.8 μm ACQUITY UPLC HSS T3 (Waters, Milford, MA, USA) column were, respectively, used for separation [31]. The column temperature and the flow rate were 60 °C and 0.4 mL/min, respectively. The detailed LC elution parameters were as follows: For ESI+ mode, the elution gradient program started from 5% B (0.1% formic acid in acetonitrile), and maintained for 0.5 min, then linearly increased to 40% B within 1.5 min and then to 100% B within 6 min, kept at 100% B for 2 min, then came back to 95% A (0.1% formic acid in water) within 0.1 min. A 2.5 min-post-equilibration was followed. For ESI-mode, the elution started from 2% B (6.5 mM ammonium bicarbonate in 95% methanol/water) and maintained for 1.5 min, then linearly increased to 40% B within 3 min, and further to 100% B within another 6 min and was kept at 100% B for 2 min, then came back to 98% A (6.5 mM ammonium bicarbonate in water) in 0.1 min. A 2 min-post-equilibration was followed. For MS acquisition, the ion source parameters for full scan were as follows: curtain gas, ion source gas 1, and ion source gas 2 were set at 35, 50, and 50 psi, respectively. Spay voltage and declustering potential (DP) were set at 5500 and 80 V, respectively. The source temperature was 500 °C. The scan range was from 50 to 800 Dalton. The accumulation time was 0.25 s. Pooled quality control (QC) samples were inserted every 10 real samples to monitor the system stability during LC-MS analysis. For information-dependent acquisition (IDA) MS/MS scan, maximum candidate ions, and intensity threshold were set at 30 and 10, with dynamic background subtraction chosen. The scan range was from 50 to 1250 Dalton. The accumulation time was 0.04 s with Zeno Pulsing on. DP was set at 80 V. The collision energy (CE) of 15, 30, and 45 V was used for different runs when QC samples were analyzed.

### 2.4. Data Processing

After data acquisition, automated peak detection from QC samples was performed using PeakView software 1.2.0.3. Then, peak annotation was performed using the home-built LC-MS^2^ database [32]. The annotation results were checked manually. Peak area integration was performed in SCIEX OS software 3.0.0.3369. Mass spectrometry responses of metabolites were normalized to the internal standards and tissue weight of their corresponding sample. The derived peak table was used for the following data exploration.

Firstly, multivariate partial least squares–discriminant analysis (PLS-DA), univariate Wilcoxon test for paired samples, and pathway analysis were performed to have an overview of the metabolic changes in GC tissues compared to the adjacent tissues. PLS-DA and pathway analysis were conducted by MetaboAnalyst 5.0 (https://www.metaboanalyst.ca/ accessed on 15 January 2021). Heatmap was used to present metabolite alterations using Multiple Experiment Viewer (MEV) software. For PLS-DA, only auto-scaling was chosen for the above manually normalized peak table in the data normalization step. No data filtration was further performed. Then, the optimal number of components for classification was determined by 5-fold cross-validation (CV). Permutation tests were performed 2000 times to validate the model. For pathway analysis, the differential metabolites, having FDR < 0.05 via univariate analysis, were input, and only the metabolites that had HMDB ID were imported. Relative betweenness centrality was chosen as the topology measure, and the hypergeometric test was chosen as an enrichment method; homo sapiens (KEGG) as the reference metabolome was used, and the minimum number of metabolites in a pathway was set as default 1. The metabolites that had a *p*-value < 0.05, as ascertained from the Wilcoxon test, were used for the mapping receiver operating characteristic curves (using the R package). The potential tissue biomarkers for gastric cancer were determined by considering both the *p*-value and area under the curve (AUC) value.

Secondly, to explore the important metabolic reprogramming of GC, differential network analysis was conducted using the R package “DGCA” [33]. Spearman correlation coefficient (SCC) was employed to evaluate the molecular correlation in each sample group. Only the metabolite pairs having significant differential correlation (False discovery rate (FDR) < 0.05) between the two groups were kept to construct the differential correlation network.

Finally, the correlations of differential metabolites with prognosis-related clinical indexes were explored. Circos plot was plotted using TBols v2.096 [34] to present the positive or negative associations of differential metabolites with the clinical indices (Mann–Whitney test, significance level defined as *p* < 0.05). A new network analysis method (NAM) was further proposed to trace the network topology changes during the progression of gastric cancer. TNM (tumor, node, and metastasis) is a well-applied staging system to describe the stages of cancer progression. A detailed NAM analysis exploring metabolic reprogramming of GC at different TNM stages was described as follows.

### 2.5. Network Topology Analysis Exploring Metabolic Reprogramming of GC at Different TNM Stages

TNM-related metabolic sub-networks were extracted using the new NAM:

*F* = {*f*_1_, *f*_2_, …, *f_m_*} represents the feature (metabolite) set, and *m* represents the number of features. The Spearman correlation coefficients between the metabolites were calculated to construct the metabolic network for each TNM stage. For two metabolites, *f_i_* and *f_j_* (1 ≤ *i* ≠ *j* ≤ *m*), *SCC_t_*(*f_i_*, *f_j_*) represents the Spearman correlation coefficient of *f_i_* and *f_j_* in TNM *t* (*t* = I, II, III). Assume that *G*_I_, *G*_II_, and *G*_III_ are the constructed networks for TNM I, TNM II, and TNM III, where the nodes represent the metabolites. Then, *G*_I_, *G*_II_ and *G*_III_ have the same node set *F*. If the two metabolites have a significant Spearman correlation (*p*-value < 0.01) in TNM *t*, then there is an edge between *f_i_* and *f_j_* in *G_t_*, and the edge weight is *w_t_*(*f_i_*, *f_j_*) = *SCC_t_*(*f_i_*, *f_j_*); otherwise, *w_t_*(*f_i_*, *f_j_*) = 0.

To study the evolution of TNM, NAM systemically analyzes *G*_I_, *G*_II_, and *G*_III_. NAM first extracts the sub-network *SG_t_* from *G_t_* (*t* = I, II, and III) based on the monotonic change change of molecular relationship during GC staging. *SG_t_* consists of the edges in *G_t_*, thereby satisfying the following conditions: (1) the edge weights monotonically decrease (or monotonically increase) in *G*_I_, *G*_II_, and *G*_III_, and (2) the edge weight difference in at least one of the two graph pairs (*G*_I_ and *G*_II_) and (*G*_II_ and *G*_III_) is larger than 0.05. Then, NAM defines the informative subnetwork triplets based on the hub nodes and their adjacent nodes in *SG_t_*.

The main procedure of NAM includes the following three steps:

Step 1. Defining the sub-network *SG_t_* ⊆ *G_t_* (*t* = I, II, and III). The node set of *SG_t_* is *F*. Let *E*(*SG_t_*) be the edge set; then,

*E*(*Temp_t_*) = {(*f_i_*, *f_j_*)|1 ≤ *i ≠ j* ≤ *m*, (*f_i_*, *f_j_*) ∈ *E*(*G_t_*), and |*w*_I_(*f_i_*, *f_j_*) − *w*_II_(*f_i_*, *f_j_*)| > 0.05 or |*w*_II_(*f_i_*, *f_j_*) − *w*_III_(*f_i_*, *f_j_*)| > 0.05};

*E*(*SG_t_*) = {(*f_i_*, *f_j_*)|1 ≤ *i ≠ j* ≤ *m*, (*f_i_*, *f_j_*) ∈ *E*(*Temp_t_*), and *w*_I_(*f_i_*, *f_j_*) ≥ *w*_II_(*f_i_*, *f_j_*) ≥ *w*_III_(*f_i_*, *f_j_*) or *w*_I_(*f_i_*, *f_j_*) ≤ *w*_II_(*f_i_*, *f_j_*) ≤ *w*_III_(*f_i_*, *f_j_*)}.

It is important to note that if the weight of an edge changes by less than or equal to 0.02 in the networks of two adjacent TNM stages, then we assume that the edge weights are almost equal in this study.

Step 2. Defining the hub nodes. The nodes in *SG_t_* were ranked according to their degree in *SG_t_*; *Hub_t_* is the set of the top 15 nodes and *Hub* = *Hub*_I_ ∪ *Hub*_II_ ∪ *Hub*_III_ is the set of important node, which is set according to the three subnetworks.

Step 3. For each important *v* node in *Hub*, an informative subnetwork triplet *Tri*(*v*) = (*Info*-*SG*_I_(*v*), *Info*-*SG*_II_(*v*), *Info*-*SG*_III_(*v*)) is defined for exploring the GC evolution, where *Info*-*SG_t_*(*v*) (*t* = I, II, and III) is the star subgraph, including the node *v* and its adjacent edges in *SG_t_*.

## 3. Results and Discussion

### 3.1. Characteristics of Enrolled GC Patients

All the eighty enrolled patients underwent a radical resection of their gastric tumor. Proximal, distal, and total gastrectomy were performed in 5, 50, and 25 cases, respectively. The clinicopathological characteristics of the enrolled GC patients are listed in Table 1. The age range of the patients was from 44 to 82 years, with a median age of 66 years. The proportion of male to female was 62/18, with males occupying 77.5% of all patients. Among the patients, 45 patients (56.3%) had a history of smoking, and 28 patients (35%) had a history of drinking. TNM I and TNM II patients accounted for 46.2% (37/80), and TNM III-IVA patients accounted for 53.8% (43/80). Most patients had no distant metastasis (76/80, 95%), and Her-2 expressions were negative (65/80, 81.3%).

### 3.2. Overview of Metabolic Alterations in GC

To evaluate the LC-MS-based metabolomics data quality, the QC samples were used for a robustness evaluation. In total, 167 metabolites were identified in the positive ionization mode, with 91.0% and 95.2% metabolites having relative standard deviations (RSDs) smaller than 20% and 30%, respectively. In total, 88 metabolites were identified in the negative ionization mode, with 92.0% and 96.6% metabolites having corresponding RSDs smaller than 20% and 30%. A total of 234 metabolites were obtained after combining the metabolites detected in both ionization modes, and they were subsequently used for data processing.

**Figure 1:**
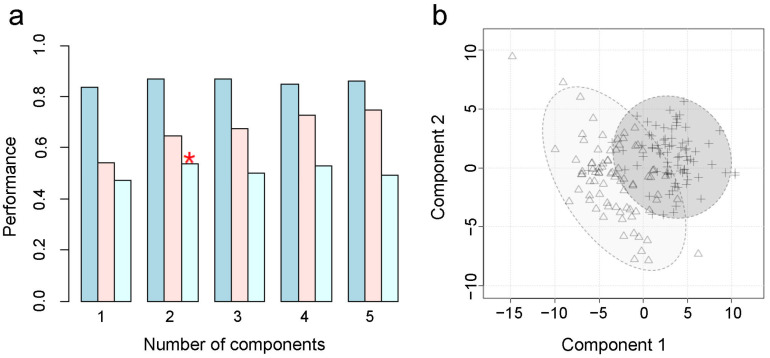
The results of partial least squares discriminant analysis. Two principal components were determined by Q2 performance (**a**). From left to right, the columns indicate the performances of accuracy, R2, and Q2. * means two principal components were kept for having the highest Q2. The score plot shows the separation between the GC tissues and adjacent non-tumor tissues (**b**). The triangles show the adjacent non-tumor tissue; the crosses show the gastric cancer tissue.

**Figure 2:**
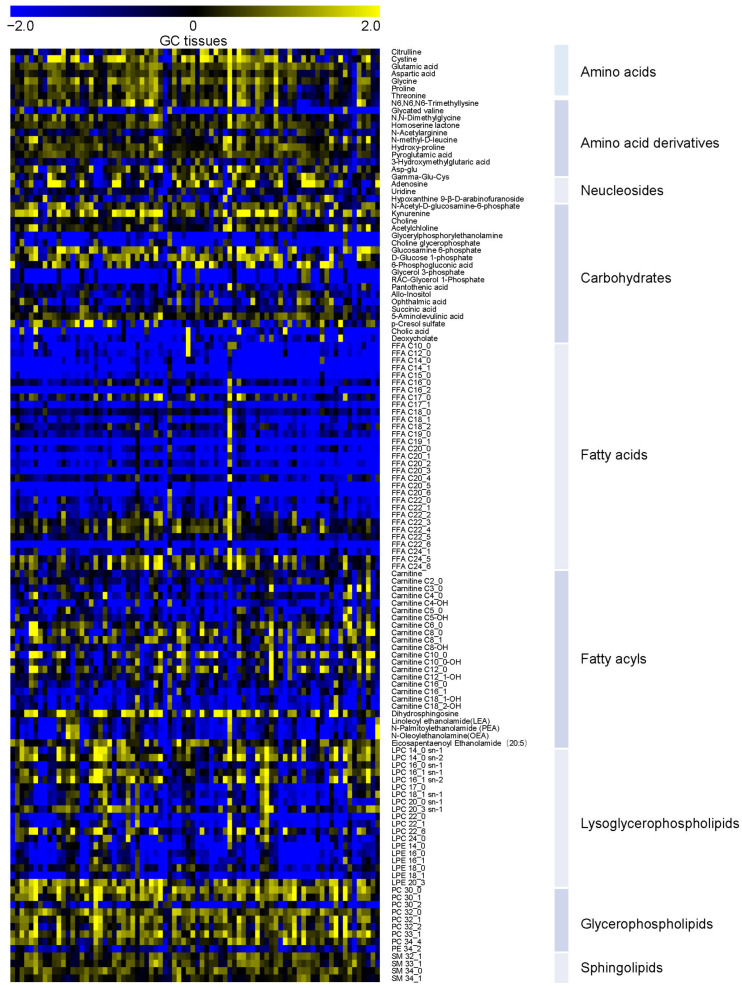
Heatmap of significantly changed metabolites screened by the nonparametric test (*p* < 0.05, FDR < 0.05).

**Figure 3:**
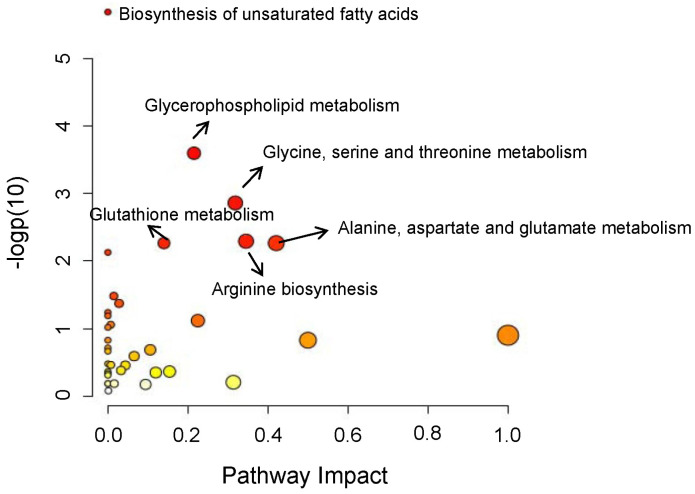
The result of pathway analysis of the significantly altered metabolites in gastric cancer tissues compared to the adjacent tissues.

To obtain an overview of the metabolic profile of GC, PLS-DA was applied. Two principal components were kept according to Q2 performance (Figure 1a), and the score plot showed an obvious separation between the GC tissues and the adjacent non-tumor tissues (Figure 1b). The model had no over-fitting when permutation tests were performed 2000 times (*p* < 0.0005). The Wilcoxon test was used for further univariate statistical analysis. The differential metabolites (*p* < 0.05) occupied 54.7% of the total metabolites. The relative abundances of differential metabolites with FDR < 0.05 are shown in Figure 2. It can be observed that there were main increases in amino acids and their derivatives, nucleosides, medium-chain acylcarnitines, glycerophospholipids, and sphingolipids, but decreases in most fatty acids and most short- and long-chain acylcarnitines. Pathway analysis was further performed, and it was found that the differentially expressed metabolites above were mainly involved in the biosynthesis of unsaturated fatty acids, glycerophospholipid metabolism, glycine, serine and threonine metabolism, glutathione metabolism, alanine, aspartate and glutamate metabolism, and arginine biosynthesis (Figure 3). Glycine [35] and proline [36] have been reported elevated in GC tissues. Glycine consumption and synthesis were strongly correlated with proliferation rates across NCI-60 cancer cell lines. Interference with the uptake of glycine and mitochondrial biosynthesis will impair cell proliferation [35]. Glutamine metabolism reprogramming is important, as it can support the tricarboxylic acid cycle, as well as nucleotide and fatty acid biosynthesis, and help maintain redox balance in cancer cells [37]. The elevated aspartate, glutamate, and glucosamine 6-phosphate may indicate that more glutamine may be absorbed and used by the tumor microenvironment. Of note, the potential GC tissue biomarker N-acetyl-D-glucosamine-6-phosphate identified by Dai et al. [19] was also significantly elevated in GC compared to their paired para-tumor tissues.

### 3.3. Potential Tissue Biomarkers for GC Diagnosis

**Figure 4:**
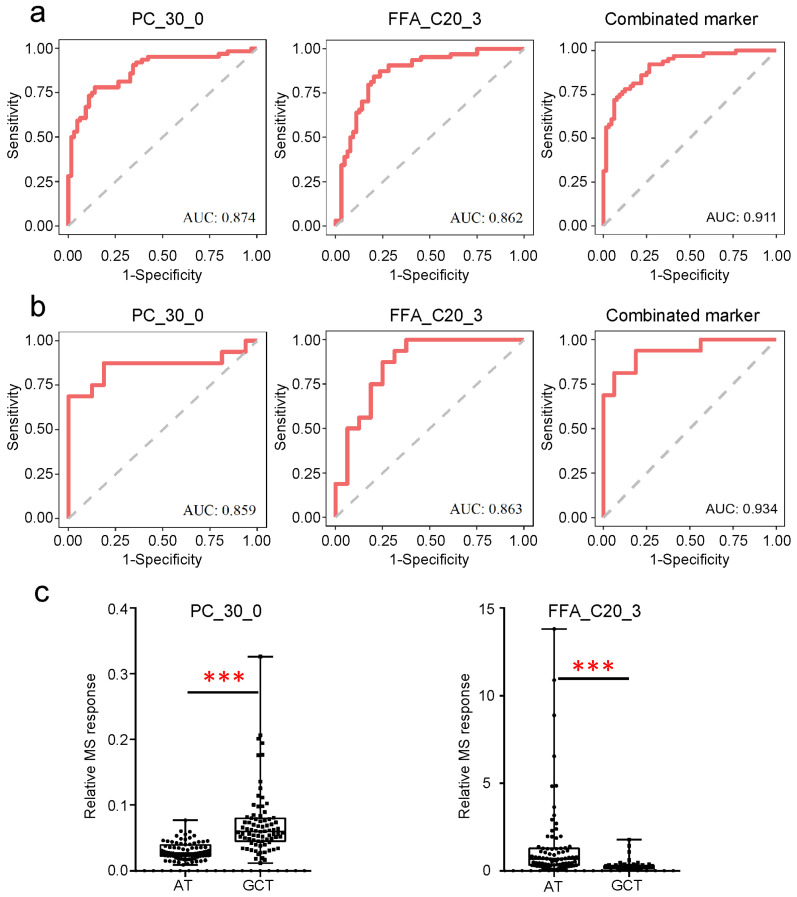
Potential tissue biomarkers for GC diagnosis. The ROC curves of PC_30_0, FFA_C20_3 and their combination in the discovery set (**a**) and the validation set (**b**). The relative MS responses of PC_30_0 and FFA_C20_3 in the discovery set (**left**, (**c**)) and the validation set (**right**, (**c**)). ***, *p* < 0.001. Boxplots indicate the median values (center lines) and IQR (box edges), with the whiskers extending to the minimum and the maximum values. IQR represents the interquartile ranges.

The tissue metabolic biomarkers can be used for rapid screening as a supplement to the established histopathologic diagnostic standard for GC. Here, potential tissue biomarkers were investigated. The samples were randomly divided into the discovery set and validation set at a 4:1 ratio. Differential metabolites (based on the discovery set) were ranked according to their *p*-values. Among the top 10 metabolites, phosphatidylcholine (PC)_30_0 and FFA_C20_3 presented good diagnosis performance, both in the discovery set and the validation set by receiver operating characteristic (ROC) curve (Figure 4a,b). PC_30_0 had AUCs of 0.874 and 0.859 in the discovery set and the validation set, respectively, and FFA_C20_3 had an AUC of 0.862 in the discovery set and an AUC of 0.863 in the validation set. PC_30_0 significantly increased, while FFA_C20_3 significantly decreased in GC tissues (Figure 4c). The combination of the two potential metabolic biomarkers had AUCs equal to 0.911 and 0.934 in the discovery set and the validation set, respectively. The sensitivities were 0.781 and 0.938 in the discovery set and the validation set, respectively, with its specificities at 0.875 and 0.813.

FFA_C20_3 showed a significant decrease in the diffuse type compared with the intestinal type, while PC_30_0 was independent of the clinical indices listed in Table A1. Tissue metabolite biomarkers for GC have been investigated less. A biomarker combining 18 metabolites has been reported differentiating malignant tissues from the adjacent non-malignant tissues of the 18 GC patients using gas chromatography–MS-based metabolomics [23]. Five metabolites, including FFA_C18:1, FFA_C18:2, and FFA_C18:3 and their derivatives have been defined as GC-associated potential biomarkers for distinguishing 30 GC tissues from paired normal tissues using LC-MS-based-metabolomic analysis. These five potential biomarkers may be dependent on the microbial metabolism to some extent [38]. FFA_C18:1 and FFA_C18:2 were also significantly decreased in GC tissues compared to paired adjacent tissues in this study (Figure 2). Thus, fatty acids as potential tissue metabolic biomarkers are worthy of further investigation using a larger patient cohort.

### 3.4. Important Fatty Acid Metabolic Reprogramming in GC

**Figure 5:**
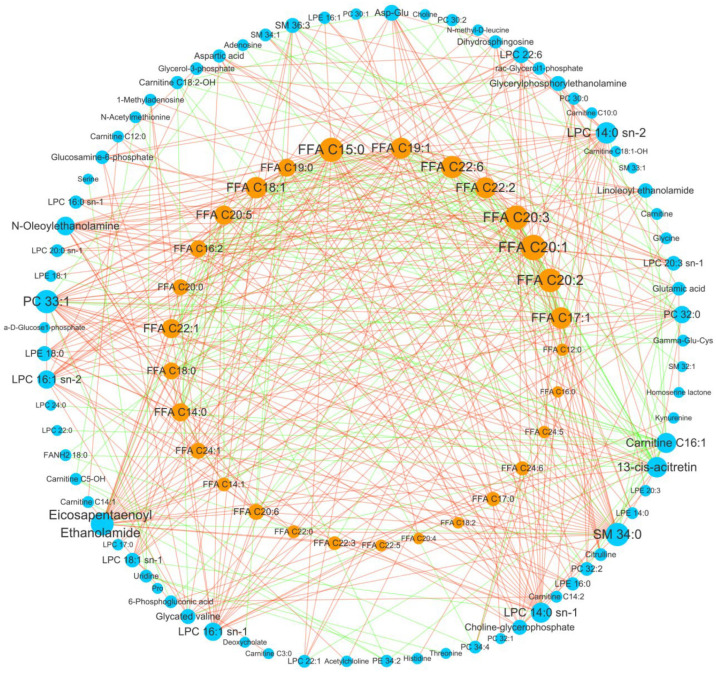
The result of differential correlation network analysis, showing intensive fatty acid metabolism-related reprogramming. Node size indicates the node degree. Edge color indicates the correlation changes from the normal group to the cancer group (red: increase; green: decrease).

To further spot important hub metabolites and metabolic pathways in GC tissue, differential correlation network analysis was performed by investigating changes in the correlation between metabolites. It showed that only one differential module was obtained after filtering the edges, with the threshold set at FDR < 0.05 (Figure 5). Within this module, the top four network nodes, as ranked by degree, were all long-chain fatty acids: FFA_C20_1, FFA_C20_2, FFA_C20_3, and FFA_C15_0 (degree larger than 28, Figure 5). The four fatty acids had a strong correlation with many other fatty acids and also with other metabolites. These correlations may indicate the importance of intensive fatty acid metabolism rewiring in GC. Most of fatty acids decreased in GC tissues, but the polyunsaturated fatty acids FFA C22_3, FFA C22_4, FFA C24_5, FFA C24_6 significantly increased in GC tissues (Figure 2). There may be an increased synthesis of polyunsaturated fatty acids in the cancer tissue microenvironment, which may be associated with tumor cell proliferation, apoptosis, and angiogenesis [22]. In addition to fatty acids, other kinds of lipids ranked after the above four polyunsaturated fatty acids were SM_34_0, PC_33_1, fatty acid ethanolamide_20_5, lysophosphatidylcholine (LPC)_14_0, and carnitine_C16_1 (degree larger than 20).

**Figure 6:**
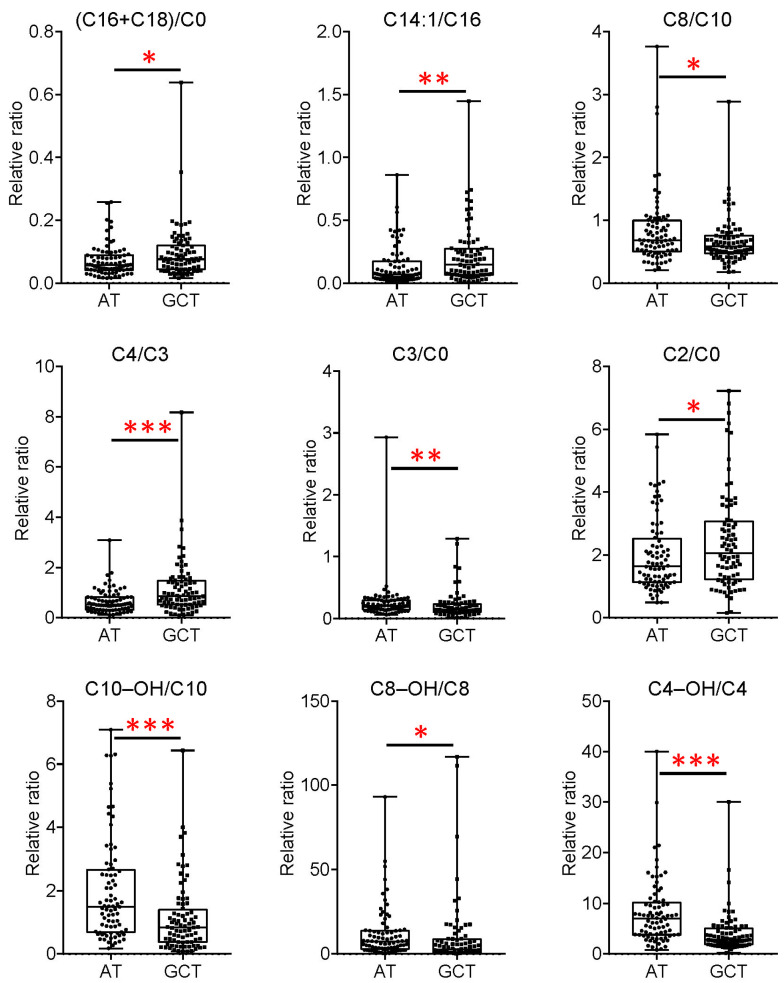
The ratios of different acylcarnitine contents in GC tissues (GCTs) and adjacent tissues (ATs). *, *p* < 0.05; **, *p* < 0.01; ***, *p* < 0.001. Boxplots indicate the median values (center lines) and IQR (box edges), with the whiskers extending to the minimum and the maximum values. IQR represents interquartile ranges.

Fatty acid β-oxidation is an important pathway for providing cellular energy during tumor progression. Acylcarnitine levels can reflect the levels of fatty acid β-oxidation in the tumor microenvironment. Though there were decreased levels of long-chain acylcarnitine, the ratio of (carnitine_C16+ carnitine_C18) to free carnitine was significantly increased in GC tissue (Figure 6), reflecting the upregulated activity of carnitine palmitoyltransferase 1 (CPT1). CPT1a has been found to have an upregulated expression in GC tissues compared to adjacent non-tumor tissue, and the situation is similar in GC cell lines corresponding with normal cells [39]. The ratios of carnitine_C14_1 to carnitine_C16 (inversely proportional to the activity of very-long-chain acyl-CoA dehydrogenase, VLCAD), carnitine_C4 to carnitine_C3 (inversely proportional to the activity of short-chain acyl-CoA dehydrogenase, SCAD), carnitine_C2 to free carnitine were significantly elevated in GC tissue (Figure 6), indicating there was an overall inhibition in even-chain fatty acid β-oxidation in GC, to some extent.

Of note, the decreased ratio of carnitine_C8 to carnitine_C10 (inversely proportional to the activity of medium-chain acyl-CoA dehydrogenase, MCAD) (Figure 6), together with the elevated medium-chain acylcarnitine, indicated the increased activity of MCAD in fatty acid β-oxidation. Inconsistent changes in MCAD expression have been reported in different types of cancers [40,41,42].

In addition, all the differential hydroxyacyl carnitines were decreased in GC tissues. The ratios of carnitine_C10-OH to carnitine_C10, carnitine_C8-OH to carnitine_C8, and carnitine_C4-OH to carnitine_C4 were significantly decreased in GC tissue (Figure 6), which may reflect the increased activity of enoyl coenzyme A hydratase short chain 1 (ECHS1). ECHS1 is responsible for the second step of mitochondrial fatty acid oxidation [43]. Its expression has been found increased in human GC cells, promoting GC cell proliferation and migration through PKB- and GSK3 beta-related signaling pathways [44]. Here, the fatty acid metabolism reprogramming was comprehensively validated in clinical GC tissues at the metabolite level, in addition to the newly found elevated medium-chain acylcarnitine and increased activity of MCAD during fatty acid β-oxidation. The exact role of the metabolites and enzymes responsible for fatty acid metabolism rewiring in GC is still not completely understood. Their influence on GC prognosis needs to be explored further.

### 3.5. The Relationships of Clinical Prognosis Indices with Differential Metabolites in GC

Various clinical physiopathological indices, such as TNM, pathological classification, molecular classification, grading, and differentiation (Table A1), are proposed to indicate complicated GC prognosis. To explore their relationships with the differential metabolites in GC, further stratified statistical analyses were performed. The derived 42 differential metabolites associated with various clinical indices were listed in Table A1, including fatty acids, acylcarnitines, fatty amides, and some amino acids and amino acid derivatives. Ranked by the number of associated metabolites, the depth of tumor infiltration and Lauren typing outranked other indices. The differential metabolites associated with infiltration depth and Lauren typing were primarily related to fatty acid metabolism.

**Figure A1:**
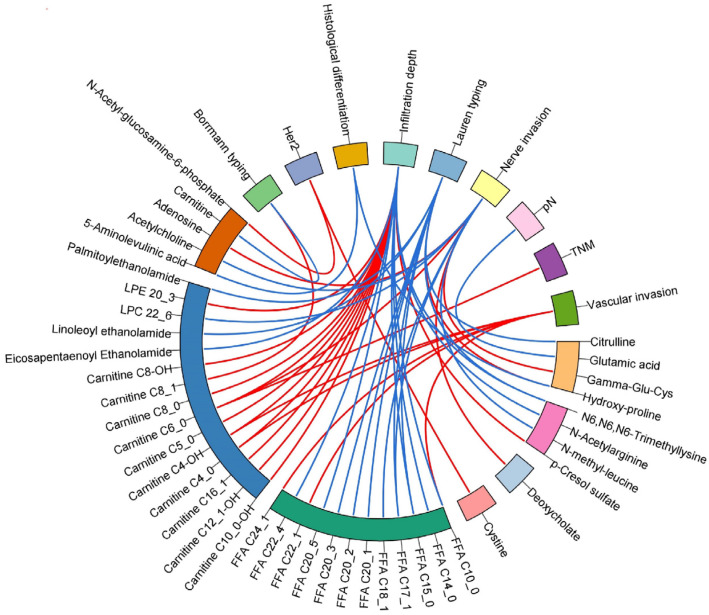
Circos plotting shows the significant associations of differential metabolites with the clinical indices. Red and blue lines show increases and decreases in metabolites, respectively, in the advanced GC tissue, which are classified by the corresponding clinical indices in Supplemental Table A1. The Mann–Whitney test was used, with the significance level defined as *p* < 0.05.

In Figure A1, Carnitine C4_0 and Carnitine C4-OH were positively correlated with vascular invasion and infiltration depth. Carnitine C6_0 was positively correlated with nerve invasion, TNM, and infiltration depth. FFA C17_1 was significantly lower in diffuse (versus intestinal) and nerve invasion (versus no nerve invasion). FFA C15_0 was significantly decreased in diffusion type (relative to intestinal type) and serosa and external organs (relative to mucosa and muscularis). In particular, carnitine C4_0 and carnitine C6_0 were significantly increased in GC (relative to adjacent tissue), and FFA C17_1 and FFA C15_0 were significantly decreased in GC, which may be potential prognosis factors related to clinical classifications. However, odd-chain fatty acids have been reported to strongly inhibit the proliferation of various human cancer cells through inhibiting activities of histone deacetylases [45].

### 3.6. TNM-Related Metabolic Network Changes

TNM system is well established to stage the cancer. To further characterize the metabolite interaction changes at different TNM stages, the metabolite–metabolite correlation network was mapped using the proposed NAM method. Two informative subnetwork triplets, *Tri*(*choline*) and *Tri*(*Carnitine C4_0-OH*), were found to have obvious alterations with TNM staging.

**Figure A2:**
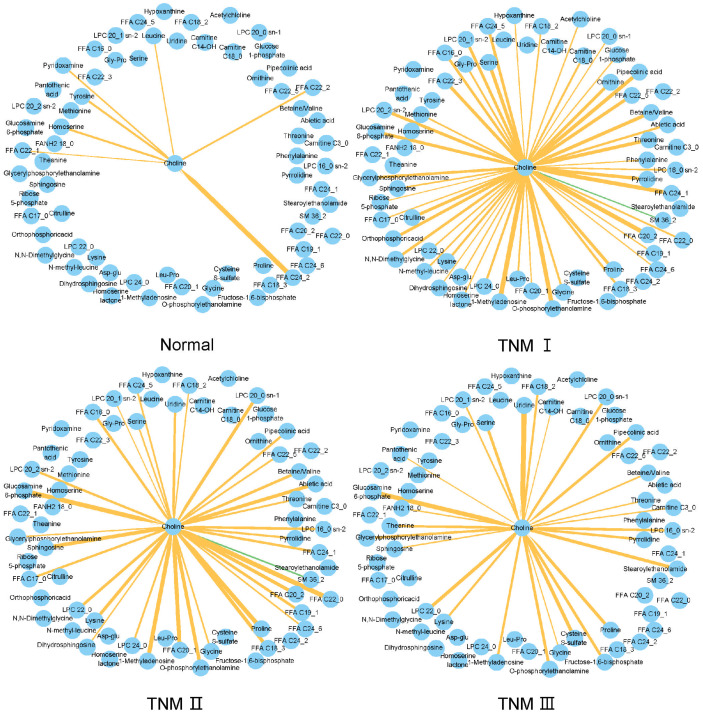
TNM-related metabolic subnetworks with choline as the hub node. Orange edges represent positive correlations, while green edges represent negative correlations between metabolites and the hub node. TNM I to TNM III were used for correlation network analysis because only four patients were diagnosed with TNM IV.

The informative subnetworks of triplet *Tri*(*choline*) in different TNM stages and the adjacent tissues are shown in Figure A2. Choline was located in the center of the networks. The levels of choline and glycerophosphorylcholine (GPC) were decreased in gastric cancer tissue in this study. In the earlier TNM I stage, choline had many positive correlations with fatty acids, LPCs, amino acids, dipeptides, etc. These positively correlated metabolites were totally different from those of normal tissue. With tumor progression, the number of correlation edges gradually decreased. From TNM I to TNM II, the correlation edges mainly with amino acids and dipeptides disappeared. From TNM II to TNM III, the correlation edges, which mostly contained fatty acids, disappeared. Choline metabolism takes part in the membrane formation, single-carbon metabolism, and cholinergic neurotransmission of normal cells [46]. Its dysregulation plays an important role in oncogenesis and tumor progression [47]. Thus, the above choline and related fatty acid metabolism need to be further explored for GC progression.

**Figure 7:**
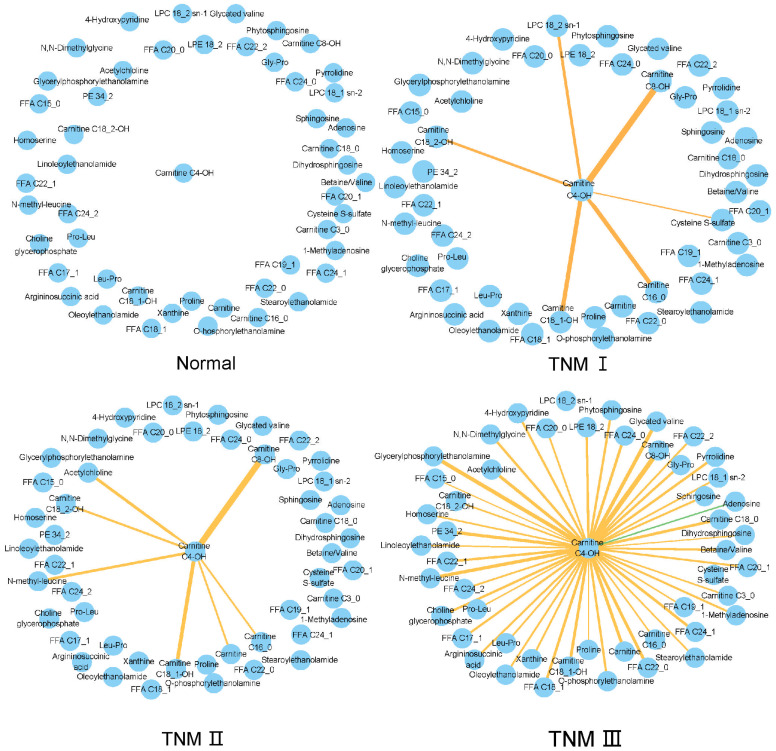
TNM related metabolic subnetworks with carnitine_C4-OH as the hub node. Orange edges represent positive correlations, while green edges represent negative correlations between metabolites and the hub node. TNM I to TNM III were used for correlation network analysis because only 4 patients were diagnosed as TNM IV.

The informative subnetworks of *Tri*(*Carnitine C4_0-OH*) in different TNM stages and the adjacent tissues are shown in Figure 7. Carnitine C4_0-OH significantly decreased in gastric cancer tissue in this study. Compared to having no correlation with surrounding metabolites in normal tissue, the number of correlation edges gradually and stably increased with GC progression. In the earlier TNM I stage, carnitine C4_0-OH had positive correlations mainly with four acylcarnitines. These four correlation edges were stable in TNM II, with new edges further built with carnitine, acetylcholine, and N-methyl-leucine. Still, the above seven correlation edges were stably kept in TNM III, and a large amount of new correlation edges were built, not only with fatty acids, glycerylphosphorylethanolamine, choline glycerophosphate, fatty acyl ethanolamine, and sphingosines, but also with amino acids and their derivatives. Tumor cells can metabolize hydroxybutyrate (one of the ketone bodies) at much higher rates than their nontumor equivalents to generate ATP as an energy source [48]. These newly increased edges in TNM III may show metabolic rewiring related to carnitine C4_0-OH, supporting the increased demand of tumor cells for energy and substances for rapid proliferation. Changes in hydroxybutyrate have been reported in the plasma and urine of gastric cancer patients [49,50]. The circulating carnitine C4_0-OH may partly reflect the tissue level of hydroxybutyrate.

Collectively, compared to the paired normal tissue, the two hub metabolites, choline, and carnitine C4_0-OH, had profound alterations when interacting with surrounding metabolites in GC tissue. The correlation edges of both hub metabolites changed gradually with GC development, with partial correlation edges robustly existing between adjacent TNM stages. These continuous alterations at the metabolic level showed metabolic rewiring during GC development, which may also suggest that the hub metabolites play a central role in tumor progression.

## 4. Conclusions

Over half of the metabolites annotated by LC-MS-based metabolomics had alterations in GC tissues compared with the adjacent tissues. There was comprehensive fatty acid metabolism reprogramming in GC tissue, with noticeable increase in levels of medium-chain acylcarnitine and activity of medium-chain acyl-CoA dehydrogenase. Of note, potential GC diagnosis biomarkers PC_30_0 and FFA_ C20_3 were identified. And TNM related metabolic network changes were defined, respectively, centered with choline and carnitine C4_0-OH. However, the present study still has some limitations. In vitro validation about the relationships between cancer cell phenotypes and key metabolites or related enzymes should be performed. Further validation for the diagnosis biomarkers in a larger cohort would be more convincing.

## Figures and Tables

**Table 1:** Clinicopathological characteristics of enrolled GC patients.

Clinical Characteristics of GC Patients (*n* = 80)	Number or Mean ± SD
Gender (No.) (male/female)	62/18
Age (years)	66.0 ± 8.3
Body mass index (BMI)	25.6 ± 22.4
Hypertension (no/yes)	54/26
Diabetes mellitus (no/yes)	69/11
Cholecystolithiasis (no/yes)	74/6
Smoking (no/yes)	35/45
Drinking (no/yes)	52/28
WHO histological classification (adenocarcinoma/signet ring cell carcinoma/mucinous adenocarcinoma)	47/19/14
Tumor differentiation (low/low–medium/medium/medium–high/high/mixed/unknown)	29/26/12/6/0/2/5
Borrmann typing (1/2/3/4/unknown)	4/41/31/2/2
Infiltration depth (mucosa (lamina propria, muscularis mucosa, and submucosa/muscularis propria/subserosa/serosa and extra serous organ)	8/13/23/36
Lauren typing (intestinal/mixed/diffuse/unknown)	21/21/30/8
Nerve invasion (No/yes/unknown)	32/42/6
Vascular invasion (no/yes/unknown)	46/28/6
pN (0/1/2/3)	27/15/17/21
pM (No/Yes)	76/4
TNM stage (I/II/III/IV)	13/24/39/4
Her2 (negative/positive)	65/15

## Data Availability

The metabolomics data have been deposited to the ProteomeXchange Consortium (http://proteomecentral.proteomexchange.org) via the iProX partner repository with the dataset identifier PXD049271.

## Appendix A

**Table A1:** Differential metabolites in gastric cancer showing associations with clinical indexes.

Differential Metabolites	*p*-Value a	FCa (GCT/AT)	Vascular Invasion b	Nerve Invasion c	Her2 Positive d	pN e	Histological Differentiation f	WHO Histological Type g	Borrmann Typing h	Infiltration Depth i	TNM j	Lauren Typing k
5-Aminolevulinic acid	***	1.3	―	―	―	―	*	―	―	―	―	―
Acetylchloline	***	1.4	―	**	―	―	―	―	―	―	―	―
Adenosine	***	2	―	**	―	―	―	―	―	―	―	―
Citrulline	*	1.3	―	―	―	―	―	―	―	―	―	*
Cystine	***	2.2	―	―	*	―	―	―	―	―	―	―
Deoxycholate	*	0.7	―	*	―	―	―	―	―	―	―	―
Gamma-Glu-Cys	*	1.3	―	*	―	―	―	―	―	―	―	―
Glutamic acid	***	1.5	―	―	―	*	―	―	―	―	―	―
Hydroxy-proline	***	1.4	―	―	―	―	**	―	―	―	―	*
N6,N6,N6-Trimethyllysine	***	1.5	―	―	―	―	―	―	―	**	―	*
N-Acetylarginine	**	0.8	―	―	―	―	―	―	―	*	―	―
N-Acetyl-glucosamine-6-phosphate	***	1.4	―	―	**	―	―	―	―	―	―	―
N-methyl-leucine	***	1.4	―	**	―	―	―	―	―	―	―	―
*p*-Cresol sulfate	**	1.2	―	―	―	―	―	―	―	*	―	―
Linoleoyl ethanolamide (18_2)	***	0.6	―	―	―	―	―	―	―	―	―	**
N-Palmitoylethanolamide (16_0)	**	0.7	―	―	―	―	―	―	―	―	―	*
Eicosapentaenoyl Ethanolamide (20_5)	***	1.4	―	―	―	―	―	―	―	*	―	―
LPC 22_6	**	1.7	―	*	―	―	―	―	―	―	―	―
LPE 20_3	***	1.9	―	―	―	―	―	―	―	*	―	―
FFA C10_0	***	0.4	*	―	―	―	―	―	―	*	―	―
FFA C14_0	***	0.2	―	―	―	―	―	―	―	*	―	―
FFA C15_0	***	0.3	―	―	―	―	―	―	―	*	―	**
FFA C17_1	***	0.3	―	*	―	―	―	―	―	―	―	**
FFA C18_1	***	0.4	―	**	―	―	―	―	―	―	―	―
FFA C20_1	***	0.2	―	―	―	―	―	―	―	―	―	*
FFA C20_2	***	0.4	―	―	―	―	―	―	―	―	―	*
FFA C20_3	***	0.2	―	―	―	―	―	―	―	―	―	**
FFA C20_5	***	0.1	―	―	―	―	―	―	―	*	―	―
FFA C22_1	***	0.4	*	―	―	―	―	―	―	―	―	―
FFA C22_4	**	1.2	―	―	―	―	―	―	―	*	―	―
FFA C24_1	**	0.6	*	―	―	―	―	―	―	―	―	―
carnitine	***	0.7	―	―	―	―	―	―	*	―	―	―
carnitine C10_0-OH	*	0.8	―	―	―	―	―	―	―	*	―	―
carnitine C12_1-OH	*	0.8	―	―	―	―	―	―	―	*	―	―
carnitine C16_1	**	0.5	―	―	―	―	―	―	―	*	―	―
carnitine C4_0	*	1	*	―	―	―	―	―	―	***	―	―
carnitine C4-OH	***	0.4	**	―	―	―	―	―	―	*	―	―
carnitine C5_0	**	0.7	―	―	―	―	―	―	―	**	―	―
carnitine C6_0	*	1.4	―	*	―	―	―	―	―	***	*	―
carnitine C8_0	*	1.8	―	―	―	―	―	―	―	*	―	―
carnitine C8_1	**	1.3	―	―	―	―	―	―	*	―	―	―
carnitine C8-OH	***	0.6	―	―	―	―	―	―	―	*	―	―

Notes: The groups for non-parameter comparisons were set as follows. a, gastric cancer tissue (GCT) vs. adjacent tissue (AT); b, vascular invasion (yes vs. no, 28/46); c, nerve invasion (yes vs. no, 42/32); d, Her2 positive (Yes vs. No, 15/65); e, pN (yes vs. no, 53/27); f, histological differentiation (low vs. medium–high, 29/18); g, WHO histological type (Signet-ring cell carcinoma and mucinous adenocarcinoma vs. adenocarcinoma, 33/47); h, Borrmann typing (3 and 4 vs. 1 and 2, 33/45); i, infiltration depth (serosa and extras serous organ vs. Mucosa and Muscularis propria, 36/21); j, TNM (III and IV vs. I and II, 43/37); k, Lauren typing (diffuse vs. intestinal, 30/21). ***, *p* < 0.001; **, *p* < 0.01; *, *p* < 0.05.
